# Cryptococcal Infection in Advanced HIV-Associated Immunosuppression—Case Series

**DOI:** 10.3390/reports9040349

**Published:** 2026-10-13

**Authors:** Isabela Ioana Loghin, Ion Cecan, Andrei Vâţă, Anton Knieling, Otilia-Elena Frăsinariu, Victor Daniel Dorobăţ, Vlad Hârtie, Carmen Mihaela Dorobăţ

**Affiliations:** 1 Faculty of Medicine, “Grigore T. Popa” University of Medicine and Pharmacy, 700115 Iasi, Romania; 2 Department of Infectious Diseases, “St. Parascheva” Clinical Hospital of Infectious Diseases, 700116 Iasi, Romania; 3 Emergency Clinical Hospital for Children “St. Maria”, 700309 Iasi, Romania; 4 National Institute for Infectious Diseases “Prof. Dr. Matei Balș”, 021105 Bucharest, Romania; 5 Department of Intensive Care, Clinical Hospital of Emergency “Prof. Dr. Nicolae Oblu”, 700309 Iasi, Romania

**Keywords:** HIV-AIDS, cryptococcal infection, cerebrospinal fluid, antifungals, meningitis, cryptococcal

## Abstract

**Background and Clinical Significance:** Cryptococcal meningitis remains a life-threatening complication of advanced HIV infection. Its recognition can be challenging when neurological manifestations are nonspecific or cerebrospinal fluid inflammation is limited. This case series illustrates the diagnostic variability and therapeutic challenges encountered in five patients managed at a regional HIV/AIDS center in northeastern Romania. **Case Presentation:** Five men aged 26–69 years were hospitalized between November 2022 and March 2025 with HIV-associated cryptococcal disease involving the central nervous system. All had profound immunosuppression, with CD4+ T-cell counts of 6–31 cells/mm^3^. Clinical manifestations included headache, confusion, altered consciousness, and focal neurological deficits. Cerebrospinal fluid opening pressures were elevated, while inflammatory findings ranged from lymphocytic pleocytosis to an almost acellular profile. Microbiological investigations included direct microscopy, fungal culture, and molecular testing, according to availability. In one patient, culture confirmed *Cryptococcus neoformans* despite negative India ink microscopy and absent documented pleocytosis. Antifungal treatment included liposomal amphotericin B and fluconazole-based regimens, with treatment selection constrained by drug availability and organ dysfunction. Three cases had fatal outcomes during hospitalization. **Conclusions:** Cryptococcal meningitis should remain a diagnostic consideration in profoundly immunosuppressed patients with neurological symptoms, even when initial microscopy is negative or cerebrospinal fluid inflammation is minimal. These cases emphasize the importance of complementary microbiological investigations, assessment and management of intracranial pressure, reliable access to recommended antifungal regimens, and appropriate timing of antiretroviral therapy.

## 1. Introduction

Cryptococcal meningitis remains a life-threatening complication of advanced human immunodeficiency virus (HIV) infection, particularly in patients with CD4+ T-cell counts below 100 cells/mm^3^ [1,2]. In Romania, national surveillance recorded 19,509 people living with diagnosed HIV/AIDS as of 30 June 2026 and 273 newly detected cases during the first half of the year, including 119 classified as AIDS [3]. The Centers for Disease Control and Prevention (CDC) surveillance classification defines adult stages 1, 2, and 3 using CD4+ thresholds of ≥500, 200–499, and <200 cells/µL, respectively. An AIDS-defining condition, including extrapulmonary cryptococcosis, establishes stage 3 regardless of the CD4+ count [4]. *Cryptococcus neoformans* is an encapsulated yeast found in soil, bird droppings, and decaying organic material. Infection usually follows inhalation, and impaired cellular immunity may allow either progression of a recent infection or reactivation of a previously contained infection, with subsequent dissemination to the central nervous system (CNS). Its capsule, melanin production, ability to grow at body temperature, and survival within macrophages contribute to pathogenicity [2]. Patients may present with headache, confusion, focal neurological deficits, or altered consciousness, while disseminated infection can produce skin lesions resembling other HIV-associated dermatoses [5,6]. Recognition may be difficult when meningeal signs and cerebrospinal fluid (CSF) inflammation are limited. Diagnostic methods offer complementary information but differ in performance, turnaround time, and cost. India ink microscopy is rapid and inexpensive; in a large validation study, its sensitivity and specificity were 86.1% and 97.3%, respectively. Culture sensitivity ranged from 82.4% to 94.2% depending on specimen volume, with 100% specificity under the study definition [7]. Culture confirms fungal viability but may take several days to two weeks [8]. Cryptococcal antigen (CrAg) lateral flow testing requires minimal equipment and provides results in approximately 10 min; CSF sensitivity and specificity were 99.3% and 99.1% in the same study [7,8]. Nevertheless, antigen excess may cause false-negative results, and persistent antigen detection after treatment does not establish fungal viability. Latex agglutination requires more laboratory processing and reagent storage resources [8]. Multiplex polymerase chain reaction can provide results in approximately one hour, although a Ugandan evaluation reported 82% sensitivity and 98% specificity against CSF CrAg, with reduced sensitivity at low fungal burdens [9]. Equipment and cartridge costs may limit access, while actual reporting times and expenditure depend on local laboratory arrangements [8,9].

Treatment requires effective antifungal induction, control of intracranial pressure, and monitoring for toxicity and drug interactions [1,10,11,12,13]. Recommended induction regimens generally combine amphotericin B with flucytosine; the WHO single-dose liposomal amphotericin B regimen also includes fluconazole [1,14]. When liposomal amphotericin B is unavailable, WHO recommends seven days of amphotericin B deoxycholate plus flucytosine, followed by seven days of fluconazole. If no amphotericin B formulation is available, fluconazole plus flucytosine for 14 days is an alternative. If flucytosine is unavailable, amphotericin B can be combined with high-dose fluconazole for 14 days [15]. Fluconazole monotherapy is less effective and remains a fallback when combination treatment cannot be provided [1,10]. These alternatives require appropriate toxicity monitoring and dose adjustment where indicated. Antiretroviral therapy (ART) is generally deferred for 4–6 weeks after antifungal treatment begins because earlier initiation can increase mortality [14,15,16,17]. Decisions should consider the clinical response, neurological stability, control of intracranial pressure, potential drug interactions, and CSF culture clearance where available. After ART initiation, neurological deterioration warrants assessment for immune reconstitution inflammatory syndrome and other causes [1,18]. CrAg screening before ART initiation or reinitiation is recommended in patients with advanced immunosuppression, particularly those with CD4+ counts below 100 cells/mm^3^ [19,20,21]. This retrospective case series describes the clinical presentations, diagnostic findings, treatment courses, and documented outcomes of five adults with HIV-associated cryptococcosis managed at a regional HIV/AIDS center in northeastern Romania.

## 2. Materials and Methods

### 2.1. Study Design and Database Information

A retrospective case series was conducted, including five adult patients with HIV-associated cryptococcosis who were evaluated and managed at the Regional HIV/AIDS Center of the “St. Parascheva” Clinical Hospital of Infectious Diseases in Iasi, northeastern Romania. Hospital medical records served as the primary source of clinical, laboratory, microbiological, imaging, therapeutic, and outcome data. The research period spanned from November 2022 to March 2025. Participants were included if they were above the age of 18 and admitted to our Regional HIV/AIDS Center in northeastern Romania after receiving an HIV diagnosis via Western blot (WB) and enzyme-linked immunosorbent assay (ELISA) testing. Clinical information was extracted retrospectively from inpatient medical records, discharge summaries, laboratory reports, microbiological results, neurological assessments, and available imaging studies. The variables collected included age and sex, HIV disease stage, CD4+ T-cell count, plasma HIV RNA level, neurological and systemic manifestations, cerebrospinal fluid characteristics, CSF opening pressure, microbiological evidence of *Cryptococcus* infection, associated opportunistic infections and comorbidities, antifungal treatment, antiretroviral therapy, opportunistic infection prophylaxis, major complications, and documented clinical outcome.

### 2.2. Ethical Approval

The “St. Parascheva” Clinical Hospital of Infectious Diseases in Iasi, Romania, granted its approval for the study on 6 February 2026 (Ethical approval no. 2/6 February 2026). Upon admittance, in the hospital protocol is mentioned that each patient signed a waiver of informed consent.

### 2.3. Study Setting

The study was conducted at the Regional HIV/AIDS Center of the “St. Parascheva” Clinical Hospital of Infectious Diseases in Iasi, a referral center for patients with HIV infection in northeastern Romania. The cases were managed between November 2022 and March 2025. Clinical care was provided in an inpatient setting, and the available records from referring institutions were reviewed when patients had undergone diagnostic evaluation or treatment before transfer. 

All blood tests were performed by the hospital’s central laboratory, and the patient’s HIV plasmatic viral load (RT-PCR HIV 1 using Cepheid’s GeneXpert® (Cepheid, CA, USA) and CD4+ T cell count were assessed by the molecular biology lab. The viral load was classified as low if undetectable or with less than 40 copies/mL, and high if detectable with more than 40 copies/mL. Microsoft Office 365 v2021 and Statistical Software for Excel (XLSTAT) version 2019 were used to conduct the statistical analysis.

## 3. Results

### 3.1. Case 1

A 69-year-old man was admitted in November 2022 with marked clinical deterioration in the setting of newly diagnosed advanced HIV infection. Before transfer to our Regional HIV/AIDS Center, he had been evaluated in a neurology department for a febrile confusional syndrome. Head CT showed no acute vascular lesion. At admission, patient was afebrile and alert but had bradyphrenia and slowed speech, together with marked dehydration. The patient was cachectic and hemodynamically unstable, with an initial blood pressure of 77/54 mmHg and peripheral oxygen saturation of approximately 89–91%.

Lumbar puncture revealed an inflammatory CSF profile. CSF analysis on 10 November 2022 showed 63 cells/mm^3^, protein 248 mg/dL (reference range: 15–60 mg/dL), glucose 20 mg/dL (reference range: 50–70), and an opening pressure of 25 cm H_2_O. Gram-stained CSF microscopy revealed numerous encapsulated yeasts, and CSF fungal culture yielded *Cryptococcus neoformans*. Antifungal susceptibility testing reported the isolate as susceptible to amphotericin B. On repeated CSF examinations, encapsulated yeasts remained detectable by microscopy. CSF leukocyte counts were 33 cells/mm^3^ on 25 November, 20 cells/mm^3^ on 13 December, 23 cells/mm^3^ on 19 December, and 27 cells/mm^3^ on 5 January 2023. The differential cell counts were predominantly lymphocytic when available. Empirical antituberculous therapy was initiated by the treating team at our Regional HIV/AIDS Center in the context of profound immunosuppression while the cause of the neurological presentation was still under investigation. *Mycobacterium tuberculosis* DNA was not detected in CSF, QuantiFERON-TB testing was negative, and the pulmonary evaluation did not support active tuberculosis. Following diagnostic reassessment, antituberculous therapy was discontinued after eight days.

The viro-immunological assessment demonstrated profound immunosuppression, with a CD4+ T-cell count of 6 cells/mm^3^ and an HIV plasma viral load of 3,510,000 copies/mL. Associated abnormalities included pancytopenia with severe anemia, electrolyte disturbances, and renal dysfunction during hospitalization. Thoracic computed tomography identified emphysematous changes and right-sided pulmonary nodular lesions, including a spiculated lesion measuring approximately 29 × 26 × 22 mm; however, the pneumology assessment did not identify findings supporting pulmonary tuberculosis.

In the setting of profound HIV-associated immunosuppression, concomitant oropharyngeal candidiasis was diagnosed by examination of a tongue scraping and initially treated with fluconazole 200 mg/day for five days, before CNS cryptococcosis was confirmed. Liposomal amphotericin B was subsequently administered at 150 mg/day (3 mg/kg/day for a body weight of 50 kg) for 32 days. Flucytosine was unavailable at the treating center during this hospitalization and therefore could not be included in the regimen. Liposomal amphotericin B was followed by fluconazole 200 mg/day for 20 days; this dose was selected because of impaired renal function, with a creatinine clearance of 50 mL/min. ART was initiated with darunavir/ritonavir in association with lamivudine/zidovudine. Trimethoprim/sulfamethoxazole was administered for *Pneumocystis jirovecii* pneumonia prophylaxis. Serial lumbar punctures were performed during the prolonged antifungal treatment.

The patient improved gradually. At discharge, he was afebrile, alert, cooperative, oriented to time and place, and hemodynamically and respiratory stable. Fluconazole maintenance therapy and continued ART were recommended, together with opportunistic infection prophylaxis and specialized follow-up.

### 3.2. Case 2

A 26-year-old man presented in March 2025 with progressive headache, clinical deterioration, confusion, right-sided motor impairment and motor aphasia, prompting emergency neurological assessment. During the initial examination, the patient was uncooperative and dysarthric, with psychomotor agitation, right palpebral ptosis, motor asymmetry, Glasgow Coma Scale score of 8 and positive meningeal signs. At initial admission, blood pressure was 90/50 mmHg, heart rate was 100 beats/min, temperature was 38.5 °C, and respiratory rate was 30 bpm. Peripheral oxygen saturation was 98% while receiving supplemental oxygen at 5 L/min via a face mask with a reservoir bag.

Cerebral CT performed on 17 March identified small hypodense lesions with an ischemic appearance involving the right mesencephalon and left lenticular/caudate region, without hydrocephalus. Brain MRI demonstrated small T1-hypointense and T2/FLAIR-hyperintense lesions involving the caudate head, capsulolenticular region, and bilateral frontoparietal white matter, with areas of diffusion restriction. Bilateral maxillary sinus retention cysts were additionally described.

Lumbar puncture performed on 18 March showed 23 leukocytes/mm^3^, with 100% lymphocytes, a glucose concentration of 42 mg/dL, a protein concentration of 148 mg/dL, and an opening pressure of 27 cm H_2_O. Direct microscopy revealed numerous encapsulated budding yeasts, establishing the diagnosis of cryptococcal meningitis. Although serum testing was compatible with late syphilis, CSF VDRL was negative.

Immunovirological assessment revealed AIDS-stage immunosuppression, with a CD4+ T-cell count of 12 cells/mm^3^ and an HIV RNA level of 30,300 copies/mL. Associated diagnoses included oropharyngeal candidiasis, splenomegaly, electrolyte abnormalities, bilateral hypoacusis, and progressive acute respiratory failure.

Empirical antibacterial therapy with meropenem and vancomycin was initiated for suspected bacterial meningitis before the CSF microscopy results became available, given the patient’s severe clinical presentation and meningeal signs. Both antibiotics were discontinued once cryptococcal meningitis was confirmed. At the time of treatment, neither amphotericin B nor flucytosine was available at the Regional HIV/AIDS Center. Consequently, antifungal induction therapy was adapted to the locally available therapeutic options and consisted of intravenous high-dose fluconazole. Fluconazole was administered intravenously at 800 mg on the first day, followed by 600 mg/day because of impaired renal function. Trimethoprim/sulfamethoxazole prophylaxis was provided. Antiretroviral therapy with bictegravir/emtricitabine/tenofovir alafenamide was initiated on 19 March during the acute episode of cryptococcal meningitis. Given the patient’s critical condition, rapid deterioration, and profound immunosuppression, the treating team prioritized prompt HIV control after weighing the perceived benefits against the risk of IRIS. Supportive neurological and intensive-care measures included oxygen therapy and management of cerebral edema.

Despite ongoing treatment, the patient’s neurological condition continued to worsen. By 27 March, he had progressed to coma with a Glasgow Coma Scale score of 3, accompanied by hemodynamic instability and severe respiratory failure requiring high-flow oxygen therapy. He subsequently developed cardiorespiratory arrest and died on 27 March 2025.

### 3.3. Case 3

The third case was a 63-year-old man transferred to the Regional HIV/AIDS Center in September–October 2024 following the diagnosis of HIV infection and cryptococcal meningoencephalitis. CSF examination showed 30 cells/mm^3^, glucose 37 mg/dL, protein 170 mg/dL, and an opening pressure of 35 cm H_2_O. Direct fungal microscopy revealed numerous encapsulated yeasts, confirming CNS cryptococcosis. Before transfer, he had presented with marked fatigue, altered mental status, and meningeal signs.

At the referring hospital, cryptococcal meningoencephalitis had been diagnosed, and intravenous fluconazole 1200 mg/day had been administered for approximately three weeks. During the same period, the patient developed an *Escherichia coli* urinary tract infection and *Enterococcus faecium* bacteremia, which were treated with linezolid and piperacillin/tazobactam. On transfer, he was conscious and cooperative but appeared pale and dehydrated, without evident meningeal signs.

Multiple nodular skin lesions were noted, some of which were ulcerated and involved the face and other anatomical sites. Cutaneous cryptococcosis was documented among the discharge diagnoses, together with the previously established cryptococcal meningoencephalitis. However, histopathological or microbiological confirmation from individual skin lesions was not available; therefore, the cutaneous involvement could not be independently confirmed.

Immunological assessment showed a CD4+ T-cell count of 31 cells/mm^3^, and plasma HIV RNA was 873,000 copies/mL. Additional findings included anemia, leukopenia, thrombocytopenia, chronic liver abnormalities, secondary syphilis, common warts involving the face and frontal region, molluscum contagiosum, and hypersplenism.

The clinical course was further complicated by infective endocarditis. Transthoracic and transesophageal echocardiography identified an echogenic mass measuring approximately 13 × 9 mm on the tricuspid valve, associated with severe tricuspid regurgitation. Subsequent echocardiographic evaluation revealed an additional lesion involving the aortic valve. *E. faecium* had previously been isolated from blood cultures. Prolonged antibacterial therapy was required, and the patient underwent further cardiological evaluation.

Fluconazole was continued following the initial high-dose course. ART was initiated with darunavir/ritonavir plus emtricitabine/tenofovir disoproxil fumarate, and trimethoprim/sulfamethoxazole prophylaxis was maintained. When transferred back to the referring infectious diseases department, the patient was afebrile, conscious, cooperative, and hemodynamically stable, although fatigue, vertigo, nausea, and reduced appetite persisted. Continued ART and prolonged antimicrobial therapy were recommended.

### 3.4. Case 4

A 40-year-old man was admitted in February 2025 after approximately five days of frontal and retro-orbital headache, chills, and marked fatigue. He had initially been evaluated for suspected sinusitis and treated with amoxicillin/clavulanate, without clinical improvement. On admission, he was conscious and oriented to time and place, afebrile, hemodynamically stable, and had no neck stiffness or other evident meningeal signs.

Lumbar puncture was initially remarkable for the paucity of inflammatory cells. The CSF was clear and contained only 2 cells/mm^3^, with an albumin concentration of 0.50 g/L. The opening pressure was 32 cm H_2_O. Microbiological examination identified *Cryptococcus neoformans*, and the isolate was reported as susceptible to amphotericin B. A repeat lumbar puncture on 6 February again showed only 2 cells/mm^3^, while direct microscopy revealed numerous encapsulated yeasts. The minimal cellular response contrasted with the abundant encapsulated yeasts seen on microscopy, in the setting of profound cellular immunodeficiency.

HIV infection was diagnosed during the same clinical episode. Immunovirological assessment revealed a CD4+ T-cell count of 10 cells/mm^3^ and HIV plasma viremia of 426,000 copies/mL. Additional diagnoses included oropharyngeal candidiasis, interstitial pneumonia, mild thrombocytopenia, hepatocytolysis, chronic serous otitis, and chronic hypertrophic rhinitis.

Caspofungin 50 mg/day was initially administered for suspected esophageal candidiasis, based on odynophagia and dysphagia in the setting of profound immunosuppression and concomitant oropharyngeal candidiasis. Ceftriaxone and dexamethasone were also administered. After one day of caspofungin, treatment was switched to fluconazole 600 mg/day because neither voriconazole nor amphotericin B was available at that time. The fluconazole dose was reduced from 1200 mg/day because of impaired renal function. ART with bictegravir/emtricitabine/tenofovir alafenamide was initiated during hospitalization, along with trimethoprim/sulfamethoxazole prophylaxis. The decision to initiate ART early reflected the treating team’s concern about profound immunosuppression and the perceived need for prompt HIV control, weighed against the risk of IRIS. On 17 February, the patient’s neurological condition deteriorated, with ataxia, somnolence, confusion, and disorientation to time and place. Emergency head CT did not reveal any acute intracranial lesion requiring neurosurgical intervention.

Repeat lumbar puncture on 18 February showed 10 cells/mm^3^ and numerous encapsulated yeasts on direct microscopy. A multiplex CSF PCR assay detected *C. neoformans*/*C. gattii*. Intravenous voriconazole 400 mg/day was initiated on 18 February while amphotericin B remained unavailable. Despite treatment, the patient’s neurological condition continued to deteriorate, and by 21 February he was markedly somnolent, with slowed speech and thought processes and minimal verbal responses, prompting intensive care evaluation and supportive management.

Liposomal amphotericin B was initiated on 27 February at 150 mg/day, corresponding to approximately 2.94 mg/kg/day for the patient’s body weight of 51 kg. Its earlier initiation had been prevented by unavailability. Later that day, the patient became profoundly unresponsive and developed tachycardia and hypoxemia, followed by cardiorespiratory arrest. He died on 27 February 2025.

### 3.5. Case 5

**Figure 1:**
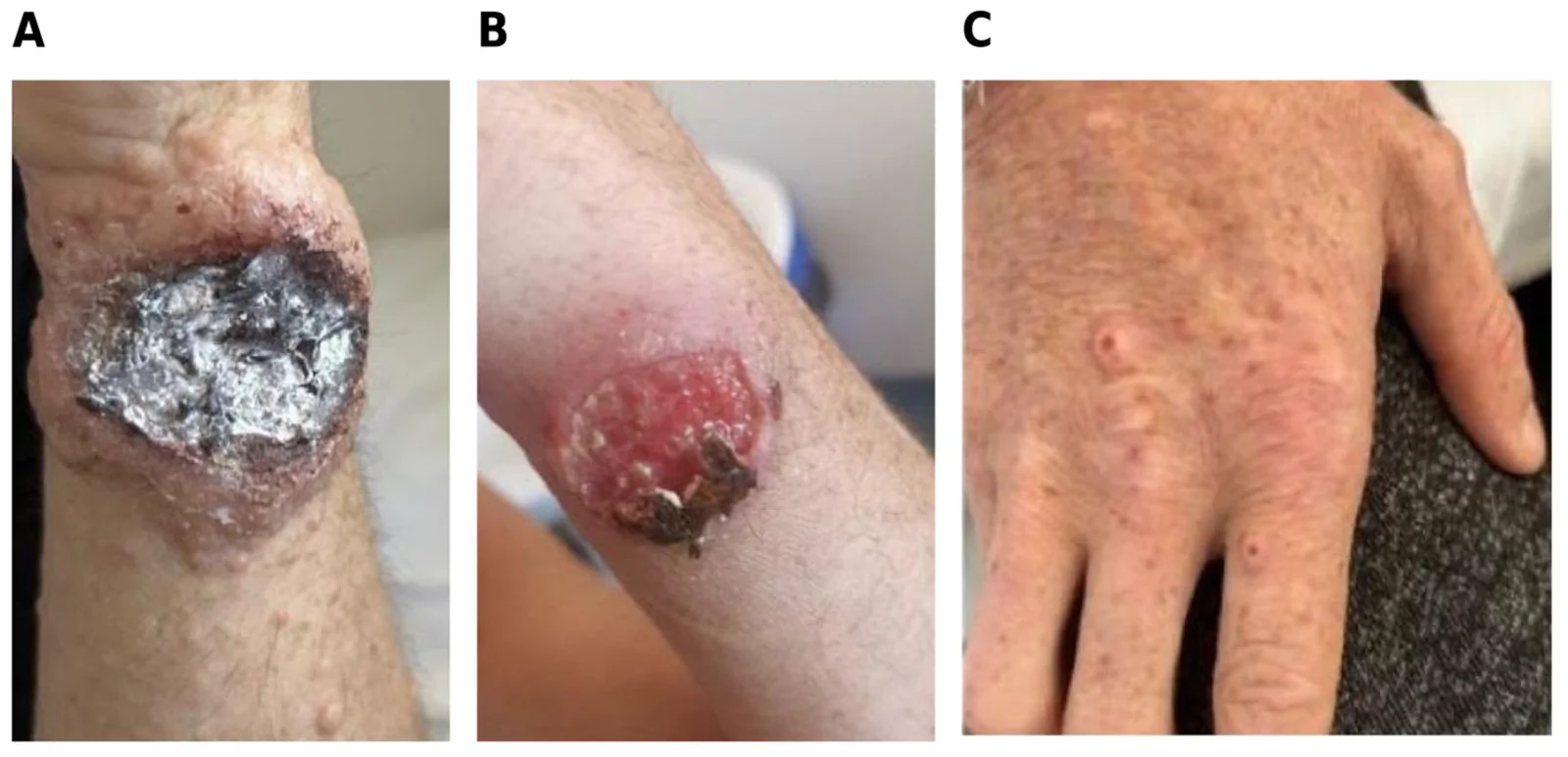
Representative cutaneous lesions observed in Case 5. (**A**) Large ulcerated lesion with crusted and necrotic components involving an extremity; (**B**) erythematous ulcerated lesion with central crusting; (**C**) multiple papular and nodular lesions involving the dorsal aspect of the hand, some displaying central umbilication. The lesions were clinically considered manifestations of disseminated cryptococcal disease; histopathological or microbiological confirmation from individual skin lesions was not available.

A 34-year-old man with advanced HIV infection was admitted with cutaneous lesions clinically suggestive of disseminated cryptococcosis and several concomitant opportunistic infections. (Figure 1A–C).

His medical history included pulmonary tuberculosis for which he was receiving antituberculous therapy and severe *Clostridioides difficile* colitis, creating a complex therapeutic context in a profoundly immunocompromised patient.

Given the diagnosis of disseminated cutaneous cryptococcosis (Figure 1A–C), CNS involvement was also investigated. Lumbar puncture was performed in October 2023, and CSF was examined by direct fungal microscopy, India ink staining, and fungal culture for *Cryptococcus*.

Biochemical analysis of CSF showed a glucose level of 49 mg/dL, albumin of 0.47 g/L, and chloride of 7.0 g/L. The concurrent blood glucose level was 90 mg/dL. The opening pressure was 33 cm H_2_O. Immunovirological assessment showed profound immunosuppression, with a CD4+ T-cell count of 22 cells/mm^3^ and plasma HIV RNA of 226,000 copies/mL (Table 1). Additional laboratory abnormalities included anemia and thrombocytopenia, together with elevated inflammatory markers: C-reactive protein reached 144.03 mg/L and the erythrocyte sedimentation rate was 140 mm/h. The HBsAg test, anti-HCV and RPR tests were negative. Fungal culture of a tongue swab was positive for *Candida albicans*.

Because the disseminated cutaneous presentation raised concern for CNS involvement, lumbar puncture was performed on 5 October 2023. The opening pressure was 33 cm H_2_O. CSF was clear and showed no inflammatory cellular response; glucose was 49 mg/dL and albumin 0.47 g/L. India ink examination was negative, whereas fungal culture subsequently yielded *C. neoformans*, confirming CNS involvement (Table 2).

Antifungal treatment consisted of intravenous voriconazole 400 mg/day was initially administered because liposomal amphotericin B was unavailable. Once available, liposomal amphotericin B was initiated at 150 mg/day (approximately 2.9 mg/kg/day for a body weight of 52 kg) (Table 3). During the subsequent clinical course, the patient developed acute respiratory failure followed by cardiorespiratory arrest. Despite maximal resuscitative measures, the patient died.

The clinical and immunovirological characteristics of the five patients are summarized in Table 1.

Given the predominance of central nervous system involvement in the present series, the available CSF and microbiological findings were analyzed separately and are presented in Table 2. Considerable heterogeneity in the CSF inflammatory response was observed, ranging from marked pleocytosis to an almost acellular profile despite direct microbiological evidence of cryptococcal infection.

Antifungal treatment, antiretroviral therapy, opportunistic infection prophylaxis, complications, and documented outcomes are summarized in Table 3.

## 4. Discussion

Cryptococcosis in this series occurred in the setting of profound HIV-associated cellular immunosuppression. All five patients had CD4+ T-cell counts below 50 cells/mm^3^, with a median of 12 cells/mm^3^, and detectable HIV RNA, with a median of 426,000 copies/mL. Current or previously established CNS involvement was documented in every patient, despite considerable variation in neurological presentation and CSF inflammation. These observations are consistent with the recognized susceptibility to disseminated cryptococcal disease in advanced HIV infection [1,2,22]. They also illustrate the clinical consequences of presentation after substantial immune deterioration, when diagnostic uncertainty, concurrent infections, and treatment limitations may complicate management.

The profound CD4+ T-cell depletion observed here resembles that reported in previous Romanian cohorts. Marincu et al. described 24 patients with AIDS-associated cryptococcal meningitis, a mean CD4 count of approximately 25 cells/µL, and six deaths [23]. Moroti et al. reported a median CD4 count of 20.5 cells/mm^3^ and 28.5% in-hospital mortality among 42 patients with CNS cryptococcosis [24]. Recent or concomitant tuberculosis was reported in ten patients in the Bucharest cohort, while pulmonary tuberculosis was documented in one patient in our series [24]. Such overlapping infections complicate diagnostic attribution and require careful coordination of antimicrobial treatment. Although all five patients in our series were men, this distribution cannot establish male sex as an independent risk factor; the sex distribution of the underlying HIV population and referral patterns must also be considered. Likewise, differences in cohort size, treatment periods, and outcome ascertainment preclude direct mortality comparisons.

Diagnostic interpretation was particularly challenging when the inflammatory response was limited. Case 5 had no documented inflammatory cellular response and negative India ink microscopy, yet CSF fungal culture yielded *C. neoformans*. This observation illustrates why negative microscopy cannot exclude cryptococcal meningitis in a severely immunocompromised patient. Cryptococcal antigen (CrAg) testing complements microscopy and culture, providing a rapid diagnostic approach [8]. In the multicentre validation study by Boulware et al., the CSF CrAg lateral flow assay had 99.3% sensitivity and 99.1% specificity, compared with 86% sensitivity for India ink microscopy [7]. Diagnostic assessment should therefore combine the clinical presentation with complementary microbiological methods, rather than interpret a negative direct examination in isolation. Follow-up results also require caution: persistently visible yeasts or abnormal CSF biochemistry do not necessarily demonstrate viable organisms or microbiological failure. Repeat culture and clinical reassessment are needed before persistent microscopy alone is used to justify prolonged or intensified treatment [1].

Cutaneous findings provided an additional reason to investigate dissemination. Suspected cryptococcal skin involvement was described in Cases 3 and 5, both with documented CNS disease. Papular, nodular, ulcerated, or centrally umbilicated lesions can resemble other opportunistic conditions; concomitant molluscum contagiosum in Case 3 complicated clinical attribution. Murakawa et al. described cutaneous involvement in patients with AIDS-associated cryptococcosis and systemic disease [5], while Ghigliotti et al. documented its resemblance to molluscum contagiosum [6]. However, neither histopathological examination nor culture of individual lesions was available in our cases. The skin findings should therefore remain clinically suspected manifestations, rather than independently confirmed cryptococcal lesions. Their practical significance was the need to assess systemic involvement.

Earlier recognition may also depend on screening before symptomatic CNS disease develops. Ford et al. reported pooled CrAg prevalences of 6.5% at CD4 counts ≤ 100 cells/mm^3^ and 2.0% at counts of 101–200 cells/mm^3^ [21]. Evidence from Mfinanga et al. and Temfack et al. supports incorporating screening and targeted pre-emptive treatment into care for advanced HIV infection [19,20]. All patients in our series already had CNS involvement when evaluated; the records cannot establish whether screening would have prevented these episodes. Nevertheless, the cases identify a clinical opportunity for timely CrAg testing, followed by investigation for meningitis when indicated, rather than assuming that antigen positivity represents isolated asymptomatic infection [1,14,17].

Raised intracranial pressure was another consistent finding, with opening pressures of 25–35 cm H_2_O and a median of 32 cm H_2_O. Pressure control is an essential component of treatment, alongside antifungal therapy. Studies by Bicanic et al., Rolfes et al., and Meda et al. support systematic pressure assessment and therapeutic lumbar punctures [11,12,13]. Rolfes et al. found an association between therapeutic lumbar puncture and improved acute survival, including among patients without markedly elevated initial pressure [12]. In our series, repeated CSF examinations were most clearly documented in Case 1, but the records did not consistently specify drainage volumes, closing pressures, or symptomatic responses. Repeated diagnostic sampling therefore cannot be assumed to represent adequate therapeutic drainage. Standardized documentation should include the opening pressure, volume removed, closing pressure, and neurological response, allowing subsequent procedures to be guided by both symptoms and measured pressure. The absence of these details limits evaluation of an intervention that may influence survival independently of antifungal selection.

The IDSA guideline, the subsequent global guideline, and EACS recommendations emphasize repeated lumbar punctures or CSF diversion when required to control intracranial hypertension [1,10,17]. EACS recommends repeated punctures until opening pressure is below 20 cm H_2_O [17]. Routine corticosteroids or osmotic agents should not replace CSF drainage [1,10]. In the randomized trial by Beardsley et al., adjunctive dexamethasone produced worse disability outcomes, more adverse events, and slower fungal clearance without a survival benefit [25]. Corticosteroids and osmotic therapy were documented in our series, but their indications and timing could not be reconstructed consistently. These limitations prevent assessment of appropriateness for individual episodes and reinforce the importance of recording the indication for each intervention.

Antifungal management should be interpreted against both the recommendations available during the study period and subsequent guidance. The 2010 IDSA guideline established amphotericin B plus flucytosine induction, followed by fluconazole consolidation and maintenance [10]. The 2024 ECMM/ISHAM/ASM global guideline, endorsed by IDSA, recommends daily liposomal amphotericin B at 3–4 mg/kg with flucytosine for induction in settings with adequate resources [1]. WHO recommends a single 10 mg/kg dose of liposomal amphotericin B combined with 14 days of flucytosine and fluconazole [14]. EACS version 13.0 likewise distinguishes daily combination induction in high-resource settings from the single-dose regimen in resource-limited settings [17]. These regimens require their accompanying drugs; a single amphotericin dose is not an equivalent standalone strategy.

The evidence supporting combination induction is clinically relevant to the limitations encountered here. In AMBITION-cm, Jarvis et al. demonstrated non-inferiority of single-dose liposomal amphotericin B with flucytosine and fluconazole, with fewer severe adverse events than the comparator regimen [26]. Ten-week mortality was 24.8% versus 28.7%, and grade 3 or 4 adverse events occurred in 50.0% versus 62.3% of participants, respectively [26]. Molloy et al. further established effective induction options for resource-limited settings [27], while Day et al. demonstrated improved survival with amphotericin B plus flucytosine compared with amphotericin B alone [28]. The latter trial reported a hazard ratio for death of 0.61 at 70 days, supporting the importance of the flucytosine component rather than merely amphotericin exposure [28]. Consolidation and maintenance remain necessary after induction. WHO recommends fluconazole 800 mg/day for eight weeks, followed by 200 mg/day maintenance [14]. EACS recommends 800 mg/day until CSF culture negativity and ART initiation, with subsequent reduction to 400 mg/day, followed by maintenance for at least 12 months and reassessment against immune recovery criteria [17].

In practice, local antifungal availability prevented consistent delivery of preferred induction regimens. Neither amphotericin B nor flucytosine was available during the hospitalization of Case 2. Cases 4 and 5 received alternative azoles before liposomal amphotericin B became available, and flucytosine was unavailable during treatment of Case 1. These circumstances explain the documented choices but do not demonstrate equivalence to recommended treatment. Where a preferred drug is unavailable, guideline-supported alternatives should be considered according to the agents actually accessible, with urgent efforts to obtain effective combination therapy [1,14,17]. The present retrospective account cannot determine whether procurement, transfer, or another therapeutic strategy would have changed individual outcomes. It nevertheless makes the timing of antifungal access an essential part of interpreting each clinical course and its outcome. Voriconazole was used under these constraints, although it is not a preferred initial induction agent [1,10]. Delayed access to effective combination therapy may have contributed to deterioration. The cases therefore identify a service limitation requiring reliable procurement and timely access to recommended antifungals, rather than evidence supporting the alternative sequences used.

In Case 4, caspofungin was administered for one day for suspected esophageal candidiasis, based on odynophagia and dysphagia. Its intended indication was distinct from the treatment of cryptococcal meningitis. Experimental studies by Moreira-Walsh et al. and Maligie and Selitrennikoff investigated mechanisms underlying cryptococcal resistance to echinocandins [29,30]; these findings do not support their clinical use against cryptococcal CNS infection. The manuscript has therefore distinguished treatment directed at suspected candidiasis from the subsequent antifungal management of cryptococcosis. No causal conclusion regarding caspofungin exposure and the fatal outcome can be drawn from this case. Dose interpretation also requires the formulation, body weight, treatment phase, and organ function. Liposomal amphotericin B at 150 mg/day corresponded to approximately 3 mg/kg/day in patients weighing 50–52 kg. The absolute dose alone therefore gives an incomplete impression of exposure, although the accompanying agents and timing remain relevant to guideline concordance. Hamill et al. found lower nephrotoxicity with liposomal amphotericin B at 3 mg/kg/day than with conventional amphotericin B [31]. In Case 1, renal dysfunction, electrolyte disturbances, anemia, and pancytopenia occurred during prolonged therapy, without establishing exclusive drug causation. Renal adjustment of fluconazole should be distinguished from selection of an appropriate dose for induction or consolidation; adjustment alone does not establish regimen adequacy. Renal function, potassium, magnesium, blood counts, and hepatic parameters should be monitored during treatment, with attention to concurrent medications and interactions. Weight-based dosing should be reported explicitly to distinguish a modest absolute dose from an inappropriately reduced dose.

The timing of ART represents a further departure from standard management. WHO and EACS recommend deferring ART for 4–6 weeks after antifungal treatment begins in cryptococcal meningitis [14,17]. EACS allows consideration of earlier initiation in selected patients when optimal treatment and very close monitoring are available [17]; these conditions should not be presumed in a series affected by antifungal shortages. The randomized COAT trial by Boulware et al. found higher 26-week mortality with ART initiated at 1–2 weeks than with initiation at approximately five weeks, particularly in patients with very low CSF leukocyte counts [15]. The systematic review by Eshun-Wilson et al. also supports caution regarding early ART [16]. In COAT, mortality was 45% with earlier ART and 30% with deferred ART, providing a stronger basis for timing decisions than an uncontrolled sequence of deterioration and treatment changes [15].

In Cases 2 and 4, the treating teams initiated ART during active CNS cryptococcosis because of clinical deterioration and concern about uncontrolled HIV infection. This rationale describes the decisions but does not establish a benefit from immediate viral suppression during acute meningitis. Both patients subsequently died, and Case 4 initially had only 2 CSF leukocytes/mm^3^. Cryptococcal immune reconstitution inflammatory syndrome (IRIS) may have contributed to deterioration, but the temporal relationship alone is insufficient for diagnosis [18]. Ingle et al. found no clear evidence of increased mortality with earlier ART in a retrospective cohort from Europe and North America; however, wide confidence intervals and unavailable IRIS incidence limit interpretation [32]. These findings cannot establish the safety of immediate ART or exclude IRIS in our patients.

Clinical deterioration after ART therefore requires consideration of persistent infection, intracranial hypertension, concurrent infections, drug toxicity, and IRIS [18,33]. The available records do not permit confident separation of these mechanisms. Three deaths occurred during the documented clinical courses: Cases 2 and 4 developed progressive neurological deterioration, whereas Case 5 died following acute respiratory failure and cardiorespiratory arrest. These terminal events do not independently establish the underlying cause of death. Jarvis et al. identified altered mental status, high fungal burden, and slower fungal clearance as determinants of early mortality [34]. Neurological deterioration was prominent in our fatal cases, but quantitative fungal burden and serial culture clearance were insufficiently documented to assess the remaining prognostic factors. The principal limitations are the small sample, retrospective design, heterogeneous records, and incomplete follow-up, particularly after transfer in Case 3. Treatment sequences, toxicity, and outcomes could be described, but comparative efficacy and causal relationships could not be evaluated. In particular, neither the number of deaths nor the apparent response of an individual survivor supports ranking the regimens administered or estimating their relative effectiveness. CrAg titers, quantitative fungal burden, serial cultures, and therapeutic drainage details were not consistently available. Clinical suspicion of cutaneous involvement could not be independently confirmed. Intermittent drug shortages further complicated interpretation because treatment selection reflected access as well as clinical judgment. The experience of severe opportunistic CNS disease in other regional HIV cohorts also supports attention to timely diagnosis and sustained engagement in care [35]. Within these limits, the series identifies practical priorities: earlier recognition, documented pressure management, dependable access to combination induction, and appropriately timed ART. These priorities should inform prospective care pathways rather than be interpreted as treatment effects demonstrated by this case series.

## 5. Conclusions

This case series highlights the diagnostic and therapeutic challenges of cryptococcal disease in advanced HIV infection and the importance of maintaining clinical suspicion despite atypical findings. A systematic differential diagnosis, supported by complementary microbiological investigations, can clarify the cause of neurological illness and guide targeted treatment. Negative direct microscopy or limited CSF inflammation should therefore prompt further investigation when the clinical presentation remains suggestive of cryptococcal meningitis. The clinical improvement observed during treatment illustrates that recovery is possible even in patients with profound immunosuppression. Supporting such recovery requires an integrated approach that combines effective antifungal therapy with management of intracranial pressure, monitoring of treatment toxicity, and appropriate timing of ART. Guideline-recommended regimens should guide treatment selection, including the choice of alternatives when preferred agents are unavailable. Reliable access to antifungal drugs and continued clinical reassessment are essential to translate these recommendations into routine care. The central clinical message is that careful diagnostic evaluation must be followed by timely, coordinated treatment. Together, these measures provide a basis for improving outcomes and limiting preventable complications. Earlier HIV diagnosis and sustained engagement in care remain important opportunities to prevent presentation with advanced opportunistic disease.

## Figures and Tables

**Table 1:** Clinical and Immunovirological Characteristics of the Five Patients with HIV-Associated Cryptococcosis.

Characteristic	Case 1	Case 2	Case 3	Case 4	Case 5
Age/Sex	69/M	26/M	63/M	40/M	34/M
HIV stage	AIDS, C3	AIDS, C3	AIDS, C3	AIDS, C3	AIDS, C3
CD4+ T cells (cells/mm^3^; reference range: 500–1400)	6	12	31	10	22
HIV RNA (copies/mL; undetectable: <40)	3,510,000	30,300	873,000	426,000	226,000
Main cryptococcal manifestation	Cryptococcal meningitis	Cryptococcal meningitis/meningoencephalitis	Cryptococcal meningoencephalitis with clinically diagnosed cutaneous involvement	Acute cryptococcal meningitis/meningoencephalitis	Disseminated cutaneous cryptococcosis with confirmed CNS involvement
Main neurological presentation	Febrile confusional syndrome, bradypsychia, bradylalia	Headache, confusion, focal motor deficit, motor aphasia, progressive impairment of consciousness	Previous confusional and meningeal syndrome	Frontal and retro-orbital headache followed by ataxia, confusion, somnolence, and severe neurological deterioration	Neurological presentation incompletely documented
Relevant comorbidities and coinfections	Cachexia, pancytopenia, renal dysfunction, electrolyte disturbances, pulmonary nodular lesions	Late latent syphilis, oropharyngeal candidiasis, splenomegaly, electrolyte disturbances, acute respiratory failure	*E. faecium* infective endocarditis, secondary syphilis, cytopenias, chronic hepatopathy, molluscum contagiosum	Oropharyngeal candidiasis, interstitial pneumonia, thrombocytopenia, hepatocytolysis	Pulmonary tuberculosis, *C. difficile* colitis, marked hepatobiliary abnormalities

**Table 2:** Cerebrospinal Fluid and Microbiological Findings in Patients with HIV-Associated Cryptococcal Disease.

Characteristic	Case 1	Case 2	Case 3	Case 4	Case 5
Initial/representative CSF leukocyte count (cells/mm^3^)	63 initially; subsequently 33, 20, 23, and 27	23 on repeat LP at our center; marked pleocytosis reported at the referring hospital	30	2 initially; 10 on subsequent LP	No inflammatory cellular response documented quantitative cell count not reported
Predominant cellular pattern	Predominantly lymphocytic on subsequent examinations	100% lymphocytes	Predominantly lymphocytic on subsequent examinations	Predominantly lymphocytic on subsequent examinations	Not applicable
CSF glucose (mg/dL) (mg/dL; reference range: 50–70)	20	42	37	30	49
CSF protein (mg/dL; reference range: 15–60)	248	148	170	145	190
CSF albumin (g/L)	Elevated on subsequent examinations	1.75	1.45	0.50	0.47
CSF opening pressure (cm H_2_O)	25	27	35	32	33
CSF chloride (g/L; reference range: 7–7.6)	6.78 g/L initially	7.00 g/L	6.00 g/L	6.80 g/L	7.00 g/L
Direct fungal microscopy	Encapsulated yeasts repeatedly observed	Numerous encapsulated budding yeasts	Numerous encapsulated yeasts	Numerous encapsulated yeasts	India ink negative; no encapsulated yeasts detected
Cryptococcus identification	*C. neoformans* identified by CSF fungal culture.	Cryptococcal meningitis established by CSF findings	*C. neoformans* identified by CSF fungal culture; CSF microscopy demonstrated encapsulated yeasts.	*C. neoformans* identified and reported susceptible to amphotericin B	CSF fungal culture positive for *C. neoformans*
Microbiological confirmation	Gram-stained CSF microscopy and positive fungal culture; isolate reported as susceptible to amphotericin B.	CSF microscopy showing numerous encapsulated budding yeasts	CSF microscopy demonstrated encapsulated yeasts, and CSF fungal culture yielded *C. neoformans*.	CSF microscopy showing numerous encapsulated yeasts; *C. neoformans* identified; multiplex PCR positive for *C. neoformans/C. gattii*	CSF fungal culture positive for *C. neoformans*; India ink negative
CNS cryptococcosis confirmed	Yes	Yes	Yes	Yes	Yes

**Table 3:** Antifungal Therapy, Antiretroviral Treatment, Opportunistic Infection Prophylaxis, and Clinical Outcomes.

Characteristic	Case 1	Case 2	Case 3	Case 4	Case 5
Initial antifungal therapy	Fluconazole 200 mg/day for 5 days	Intravenous fluconazole 800 mg on the first day.	IV fluconazole 1200 mg/day for approximately 3 weeks before transfer	Caspofungin 50 mg/one day Fluconazole 600 mg/day	Voriconazole 400 mg/day IV;
Subsequent antifungal therapy	Liposomal amphotericin B 3 mg/kg/day for 32 days, then renal-adjusted fluconazole 200 mg/day for 20 days.	Fluconazole 800 mg IV on the first adjusted day, followed by 600 mg/day IV because of renal impairment	Fluconazole continued after transfer	Voriconazole 400 mg/day IV; liposomal amphotericin B 150 mg/day subsequently initiated	Liposomal amphotericin B 150 mg/day (approximately 2.9 mg/kg/day), initiated once available.
Amphotericin B documented	Yes	No	No	Yes	Yes
ART regimen	DRV/r + 3TC/ZDV	BIC/FTC/TAF	DRV/r + FTC/TDF	BIC/FTC/TAF	DOR/3TC/TDF
ART during active CNS cryptococcosis	Initiated during hospitalization; exact interval not established	Yes; BIC/FTC/TAF initiated on 19 March 2025	ART initiated after prior antifungal treatment; exact interval not established	Yes	Timing relative to the cryptococcal episode not documented
TMP/SMX prophylaxis	Yes	Yes	Yes	Yes	Yes
Major complications during the documented episode	Renal dysfunction, electrolyte abnormalities, pancytopenia, severe anemia	Progressive neurological deterioration, coma, acute respiratory failure	*E. faecium* infective endocarditis and multiple systemic comorbidities	Progressive neurological deterioration and severe terminal hypoxemia	Pulmonary TB, *C. difficile* colitis, severe hepatobiliary abnormalities
Final documented outcome	Clinical improvement; discharged alive	Death	Alive at transfer	Death	Death

## Data Availability

The original contributions presented in this study are included in the article. Further inquiries can be directed to the corresponding author.

## Abbreviations

The following abbreviations are used in this manuscript:

AIDS	Acquired immunodeficiency syndrome
ART	Antiretroviral therapy
CDC	Centers for Disease Control and Prevention
CNS	Central nervous system
COAT	Cryptococcal Optimal ART Timing
CrAg	Cryptococcal antigen
CSF	Cerebrospinal fluid
EACS	European AIDS Clinical Society
ELISA	Enzyme-linked immunosorbent assay
FLAIR	Fluid-attenuated inversion recovery
HIV	Human immunodeficiency virus
IDSA	Infectious Diseases Society of America
IRIS	Immune reconstitution inflammatory syndrome
MRI	Magnetic resonance imaging
NIH	National Institutes of Health
WB	Western blot
WHO	World Health Organization
